# GRSF1 suppresses cell senescence

**DOI:** 10.18632/aging.101516

**Published:** 2018-08-07

**Authors:** Ji Heon Noh, Kyoung Mi Kim, M. Laura Idda, Jennifer L. Martindale, Xiaoling Yang, Kotb Abdelmohsen, Myriam Gorospe

**Affiliations:** 1 Laboratory of Genetics and Genomics, National Institute on Aging Intramural Research Program, National Institutes of Health, Baltimore, MD 21224, USA; * Equal contribution

**Keywords:** mitochondrial dysfunction, cellular senescence, DNA damage response, IL6 secretion

## Abstract

A prominent phenotype triggered by the loss of mitochondrial homeostasis is cellular senescence, characterized by cessation of growth and a senescence-associated secretory phenotype (SASP). We identified the G-rich RNA sequence-binding factor 1 (GRSF1) as a major mitochondrial protein implicated in this response. GRSF1 levels declined in senescent cells through reduced protein stability, and lowering GRSF1 abundance caused mitochondrial stress leading to elevated production of superoxide, increased DNA damage foci, and diminished cell proliferation. In addition, reducing GRSF1 increased the activity of a senescence-associated β-galactosidase (SA-β-gal) and the production and secretion of the SASP factor interleukin 6 (IL6). Together, our findings indicate that the decline in GRSF1 levels during cellular senescence contributes to impairing mitochondrial function, elevating ROS and DNA damage, suppressing growth, and implementing a pro-inflammatory program.

## Introduction

Mitochondria are eukaryotic organelles essential for generating energy. Mitochondrial homeostasis is controlled through gene expression programs governed by mitochondrial (mt)DNA and nuclear DNA. The RNA-binding protein (RBP) G-rich sequence-binding factor 1 (GRSF1) is encoded by nuclear DNA, translated in the cytosol, and directed to mitochondria through a mitochondria-localization signal [1]. GRSF1 is a constituent of mitochondrial RNA granules, large ribonucleoprotein (RNP) structures wherein RNase P processes precursor polycistronic mitochondrial RNAs to release mRNAs and tRNAs [1]. GRSF1 subsequently enables the loading of mature mRNA onto mitochondrial ribosomes [2]. Recently, GRSF1 was found to associate with the nuclear DNA-encoded long noncoding (lnc)RNA *RMRP* in the mitochondrial matrix, thereby enhancing mitochondrial mitochondrial structure and function [3]. These studies provided molecular evidence that GRSF1 RNPs critically maintain mitochondrial function.

As the major producers of cellular ATP and reactive oxygen species (ROS) and key regulators of calcium signaling and apoptosis, mitochondria vitally affect cell homeostasis [4]. These functions directly impact upon tissue and organ physiology, influencing major processes such as innate immunity and stem cell activity. The pleiotropic actions of mitochondria provide an important framework to explain the physiologic decline and the diseases that rise with human aging. Accordingly, losses in mitochondrial structure and function have been associated with normal aging and with many age-related diseases, including neurodegeneration, cancer, diabetes, and cardiovascular disease [5].

In light of recent evidence that GRSF1 was required for maintaining mitochondrial function [3], we set out to study comprehensively the effectors of GRSF1 actions in mitochondria. We found that loss of GRSF1 triggered DNA damage and impaired cell proliferation. These responses recapitulated the phenotype of senescent cells, which arise following sublethal damage like telomere erosion, metabolic or oxidative stress, and oncogene activation [6]. Senescent cells express distinct proteins, including a senescence-associated (SA) β-galactosidase that is often used as a senescence marker, and secrete many proteins, including pro-inflammatory cytokines, matrix metalloproteases and growth factors that comprise the senescence-associated secretory phenotype (SASP) [7]. A direct role for mitochondria in senescence has been established, and clearance of mitochondria suppressed cellular senescence [8]. Our results indicate that GRSF1 prevents premature senescence by preserving mitochondrial function; accordingly, silencing GRSF1 elicited mitochondrial stress, in turn triggering DNA damage, growth suppression, increased expression of senescence markers, and heightened production of SASP factor IL6.

## RESULTS

### GRSF1 levels decline in senescent cells

**Figure 1:**
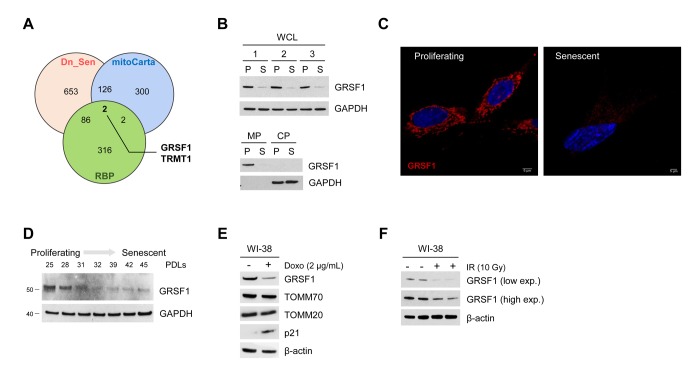
**GRSF1 levels decline with senescence.** (**A**) Venn diagram showing the overlap of mitochondrial RNA-binding proteins (overlap between mitoCarta and RBPs) that were significantly downregulated during replicative senescence (Dn_Sen). (**B**) GRSF1 protein levels were assessed in proliferating (P) and senescent (S) WI-38 fibroblasts in both WCL and in membrane (MP) and cytosolic (CP) fractions. (**C**) Representative image of GRSF1 immunofluorescence in P (population doubling level 23, PDL23) and S (PDL 58) WI-38 fibroblasts. (**D**) WI-38 cells of increasing PDLs were collected, and the levels of GRSF1 were assessed by Western blot analysis. (**E,F**) WI-38 fibroblasts (PDL23) were rendered senescent via different methods: by treatment with Doxorubicin (2 µg/mL) for 24 h followed by culture for an additional 7 days (**E**) and by exposure to 10 Gy of ionizing radiation (IR) followed by culture for 10 days (**F**). The indicated proteins were assessed by Western blot analysis.

The computational merging of proteins less abundant in the membrane fraction of senescent cells (Dn_Sen) [9], mitochondria-resident proteins (mitoCarta) [10], and RNA-binding proteins (RBPs) (http://rbpdb.ccbr.utoronto.ca/) [11] revealed that GRSF1 and tRNA methyltransferase 1 (TRMT1) were the only mitochondria-residing RBPs reduced in senescent fibroblasts (Fig. 1A). We confirmed the decline in GRSF1 levels in a model of replicative senescence: human WI-38 fibroblasts that were either proliferating (‘P’, early population doubling level, PDL23) or were rendered senescent (‘S’) by replicative exhaustion (PDL58). The levels of GRSF1 were assayed in whole-cell lysates (WCL), membrane preparations (MP), and cytosolic preparations (CP) obtained from P and S WI-38 cells by Western blot analysis (Fig. 1B). Immunofluorescence analysis confirmed that GRSF1 levels declined in senescent cells (Fig. 1C) and Western blot analysis further established that GRSF1 levels decreased early in senescence (Fig. 1D). In addition, we measured GRSF1 in cells rendered senescent by exposure to DNA-damaging agents, doxorubicin (Doxo, 2 μg/mL for 24 h) or ionizing radiation (IR, 10 Gy), and assayed 7 or 10 days later, respectively [9]. As shown, GRSF1 levels declined markedly in these senescence models (Fig. 1E,F).

### GRSF1 levels decline with senescence due to reduced protein stability

**Figure 2:**
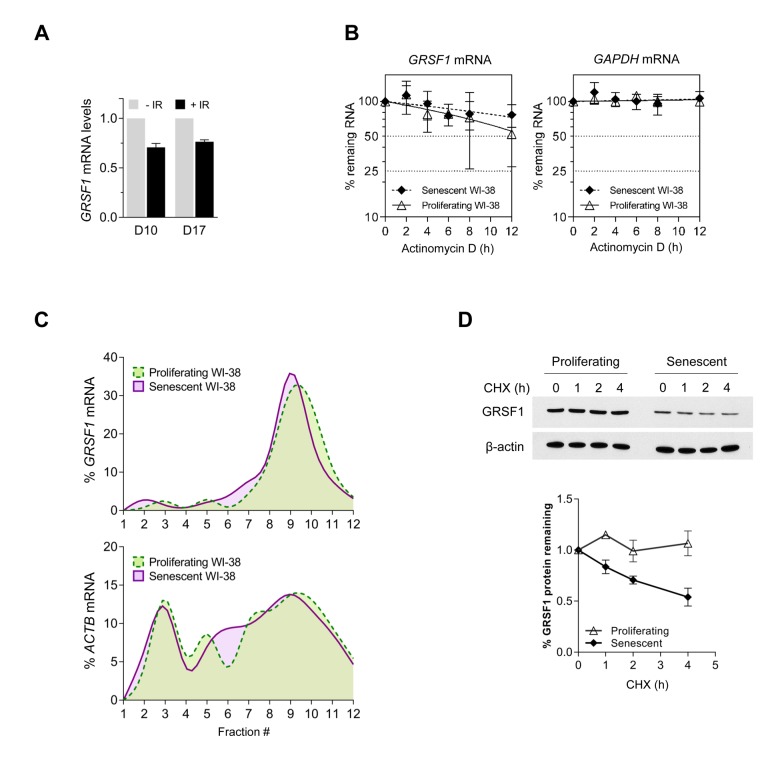
**GRSF1 protein is unstable in senescent cells.** (**A**) WI-38 fibroblasts were rendered senescent by exposure to 10 Gy of ionizing radiation (IR) followed by incubation for 10 days. Total RNA was then extracted and RT-qPCR analysis was used to measure *GRSF1* mRNA levels and *18S* rRNA levels (for normalization). (**B**) The stability of *GRSF1* mRNA was studied by incubating proliferating (PDL23) and senescent (PDL55) WI-38 fibroblasts with actinomycin D and measuring the rate of clearance of *GRSF1* mRNA (and *GAPDH* mRNA, a stable mRNA encoding housekeeping protein GAPDH) by RT-qPCR analysis; mRNA levels were normalized to *18S* rRNA levels, quantified in the same samples. (**C**) GRSF1 translation was compared between proliferating (PDL23) and senescent (PDL55) WI-38 fibroblasts by fractionating polysomes and assessing throughout the sucrose gradients the distribution of *GRSF1* mRNA and *ACTB* mRNA [encoding the control protein ACTB (β-Actin) and chosen because it is not generally subject to translational control]. Fractions 1 and 2 were devoid of ribosome particles, fractions 3-5 contained ribosomal subunits and monosomes, fractions 6-8 contained low-molecular weight polysomes, and fractions 9-12 contained high-molecular-weight polysomes (not shown). (**D**) The relative stability of GRSF1 protein in proliferating and senescent WI-38 fibroblasts was measured following incubation with 100 µg/mL of cycloheximide to block *de novo* protein synthesis. Lysates were prepared at the times shown and Western blot analysis was used to assess the levels of GRSF1 and β-Actin; after quantification by densitometry, the relative signal intensities were plotted. Data in (**A**,**D**) represent the means and S.D. from two independent experiments. Data in (**B**) represent the means and S.D. from three independent experiments.

To address whether *GRSF1* mRNA levels change during cellular senescence, we measured *GRSF1* mRNA abundance by reverse transcription (RT) followed by real-time, quantitative (q) PCR analysis after exposure to ionizing radiation (IR) with 10 Gy and additional culture for 10 or 17 days (D10, D17). Unexpectedly, only a modest reduction in the steady-state levels of *GRSF1* mRNA was observed in senescent cells (Fig. 2A). Analysis of *GRSF1* mRNA stability by treating P and S cells with actinomycin D to block *de novo* RNA synthesis, and then assessing the rate of clearance of *GRSF1* mRNA (or a stable control transcript, *GAPDH* mRNA) confirmed that *GRSF1* mRNA is highly stable in both proliferating and senescent WI-38 cells (Fig. 2B).

Therefore, we asked whether GRSF1 protein production or degradation might instead be regulated during senescence. GRSF1 protein translation was studied by fractionating the polysomal compartment of the cell on sucrose gradients using standard procedures [12]. We isolated fractions that were devoid of ribosome particles (fractions 1 and 2), fractions containing ribosomal subunits and single ribosomes (fractions 3-5), low-molecular weight polysomes (fractions 6-8), and high-molecular-weight polysomes (fractions 9-12). RNA was prepared from each fraction, and the levels of *GRSF1* mRNA in each fraction were calculated as a percentage of total *GRSF1* mRNA in the gradient. This analysis revealed that the association of *GRSF1* mRNA on polysomes of proliferating and senescent cells was rather similar, suggesting that the rate of GRSF1 translation was not strongly affected by senescence (Fig. 2C). We thus examined whether GRSF1 protein stability might differ between proliferating and senescent cells by measuring GRSF1 protein half-life in WI-38 fibroblasts following treatment with cycloheximide (CHX), an inhibitor of *de novo* translation. Interestingly, the stability of GRSF1 was markedly reduced in senescent cells relative to proliferating cells, as determined by Western blot analysis (Fig. 2D). In sum, GRSF1 levels decline in senescent WI-38 fibroblasts due in large part to the decreased stability of GRSF1 protein in senescence.

### Loss of GRSF1 increases DNA damage and suppresses cell proliferation

**Figure 3:**
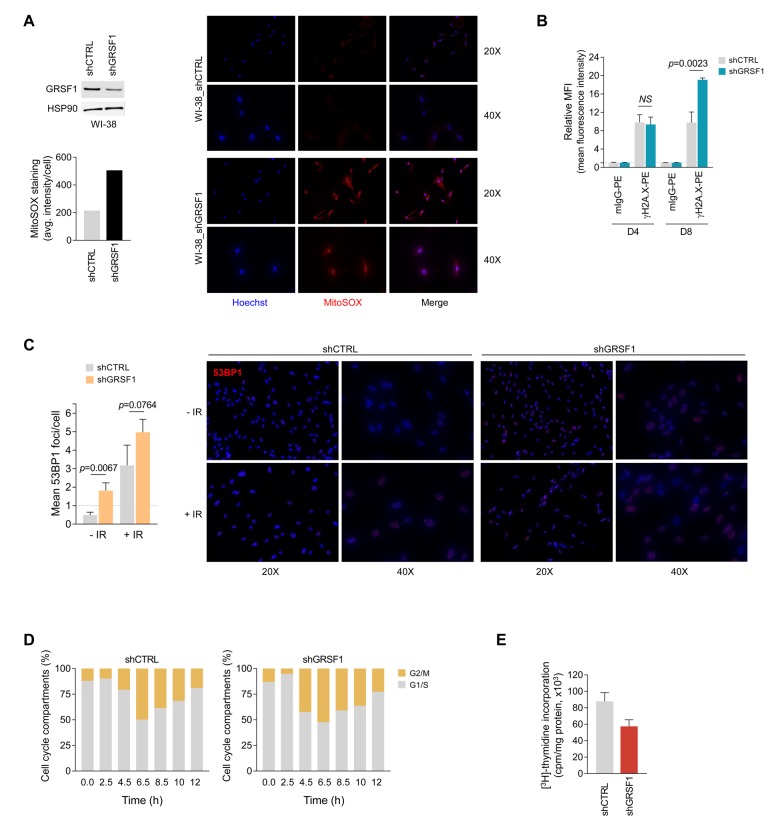
**GRSF1 knockout or silencing elevates DNA damage, reduces DNA replication, and delays cell cycle progression.** (**A**) Mitochondrial superoxide was assessed by fluorescent microscopy in shRNA-transfected WI-38 fibroblasts expressing normal (shCTRL) or reduced (shGRSF1) levels of GRSF1. The mitochondrial superoxide probe MitoSOX Red was employed to measure the relative production of superoxide, which was quantified as the average (avg.) fluorescence intensity (*left*); representative fluorescence images of the oxidized MitoSOX fluorescence signal (red) are shown (*right*). GRSF1 levels in infected WI-38 cells were assessed by Western blot analysis (*left*). (**B**) DNA double-strand breaks (DSBs) were monitored by measuring the phosphorylated histone H2A.X on serine 139 (γH2A.X) by using flow cytometry-based analysis at 4 and 8 days (D4 and D8) after IR (10 Gy). (**C**) WI-38 fibroblasts were infected with lentiviruses expressing shGRSF1 or shGRSF1; following ionizing irradiation (10 Gy) DNA damage was assessed by immunofluorescence to visualize 53BP1 foci (*right*); foci were counted in WI-38 fibroblasts expressing shCTRL or shGRSF1 that had been left untreated (-IR) or had been exposed to IR (+IR) (*Left*). (**D**) WI-38 fibroblasts that expressed constitutively shGRSF1 or shCTRL were synchronized at G1/S phase by double-thymidine block, released, and collected for analysis of cell cycle progression at the indicated time points by flow cytometry; the progression of cells through the division cycle was quantified (G2/M, G1/S). (**E**) WI-38 fibroblasts were infected with shGRSF1 or shCTRL lentiviruses and expanded for three weeks; the incorporation of [^3^H]-thymidine was then quantified as a measure of cell proliferation. Data in (**B**,**C**,**E**) represent the means and S.D. from three independent experiments.

Given that GRSF1 is needed to maintain mitochondrial oxidative phosphorylation, particularly the function of complex I [3], we asked if the senescence-associated reduction of GRSF1 might increase oxidative stress in the cell by elevating mitochondria-generated reactive oxygen species (ROS). To test this possibility, we transduced WI-38 cells with lentiviruses that expressed a GRSF1-directed (shGRSF1) shRNA to reduce GRSF1 levels constitutively or a control shRNA (shCTRL) (Fig. 3A, *top left*). The levels of mitochondria-derived superoxide, as assessed by staining with MitoSOX dye, revealed that lowering GRSF1 increased MitoSOX signals, indicating that reducing GRSF1 increased oxidative stress (Fig. 3A). To address whether the rise in oxidative damage might eventually lead to DNA damage, we asked if GRSF1 levels influenced two measures of DNA damage; both the quantification of γ-H2A.X-positive signals by flow cytometry (MFI, mean fluorescence intensity) (Fig. 3B) and the counting of immunofluorescent foci per cell that were reactive to the DNA damage response factor 53BP1 (Fig. 3C) revealed that GRSF1-silenced cells displayed increased DNA damage. Exposure to IR indicated that silencing GRSF1 further sensitized cells to DNA damage (Fig. 3C). Furthermore, WI-38 fibroblasts that were synchronized by double thymidine block and were stimulated to re-enter the cell division cycle showed a delay in cell cycle progression and transition through G2/M if GRSF1 levels were reduced (Fig. 3D). Finally, GRSF1-silenced WI-38 fibroblasts displayed lower ^3^H-thymidine incorporation (Fig. 3E). Collectively, these data indicate that loss of GRSF1 triggered mitochondria stress associated with key senescence traits, including elevated DNA damage (risen γ-H2A.X levels and 53BP1-positive foci) and reduced cell division.

### Loss of GRSF1 increases IL6 production

**Figure 4:**
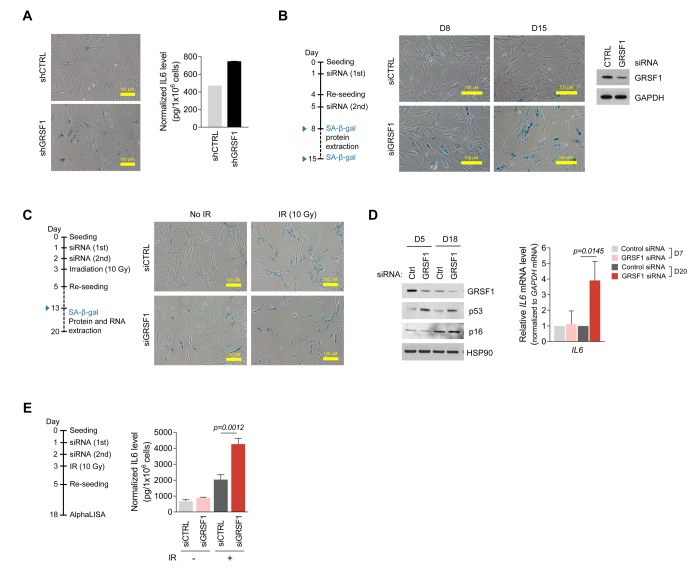
**Silencing GRSF1 promotes senescence and IL6 production.** (**A**) Three weeks after silencing GRSF1 by infection with shGRSF1- or shCTRL-expressing lentiviruses, SA-β-gal activity was assessed by light microscopy (*left*) and secreted IL6 was quantified by ELISA (*right*). **(B)** Pre-senescent WI-38 fibroblasts were transfected with siGRSF1 or siCTRL; SA-β-gal activity (micrographs) was assessed at day 8 (D8) and D15 after transfection. At D8, GRSF1 and GAPDH levels in WCL were assessed by Western blot analysis (*right*). **(C,D)** WI-38 cells transfected with siGRSF1 or siCTRL were irradiated (IR, 10 Gy) and subsequently cultured for 10 days; whereupon SA-β-gal activity was assessed (**C**); proteins in WCL prepared at D5 and D18 were assessed by Western blot analysis (**D**, *left*). RNA was extracted at D7 and D20, and *IL6* mRNA levels were quantified by RT-qPCR analysis (**D**, *right*). (**E**) After transfection with siGRSF1 or siCTRL and irradiation, WI-38 cells were cultured in normal medium for 14 days and in serum-free condition for 24 h (*left*). CM was then collected, and the secreted IL6 was assayed by AlphaLISA (*right*). Data in (**D**,**E**) represent the means ±S.D. from three independent experiments.

To investigate if the senescence-associated loss of GRSF1 also elevated the production and secretion of the major SASP factor interleukin 6 (IL6), we tested the effect of GRSF1 on IL6 abundance in several conditions. First, lentiviruses expressing shCTRL or shGRSF1 were used to silence GRSF1 in WI-38 fibroblasts Fig. 3A) and the levels of secreted IL6, as measured in conditioned medium by ELISA, were found to increase after GRSF1 silencing (Fig. 4A). Second, WI-38 fibroblasts at PDL30 were transfected with either control siRNA (siCTRL) or GRSF1-directed siRNA (siGRSF1) on days 1 and 5 and cultured for an additional 8 and 15 days (D8, D15) to allow GRSF1 levels to change. SA-β-gal analysis revealed greater numbers of positive (blue) senescent cells after GRSF1 depletion, supporting the notion that lowering GRSF1 promoted cell senescence (Fig. 4B). Third, exposure to IR triggered WI-38 fibroblast senescence regardless of GRSF1 abundance, but SA-β-gal-positive senescent cells increased after depletion of GRSF1 (Fig. 4C). Interestingly, increases in senescent marker p53 (Fig. 4D, *left*) were dependent on GRSF1 silencing. The levels of *IL6* mRNA and secreted IL6 were significantly higher in GRSF1-silenced WI-38 fibroblasts, as determined by RT-qPCR analysis (Fig. 4D) and AlphaLISA (Fig. 4E). Together, these data indicate that silencing GRSF1 accelerates senescence and induces IL6 secretion.

### Paracrine IL6 signaling following GRSF1 depletion

**Figure 5:**
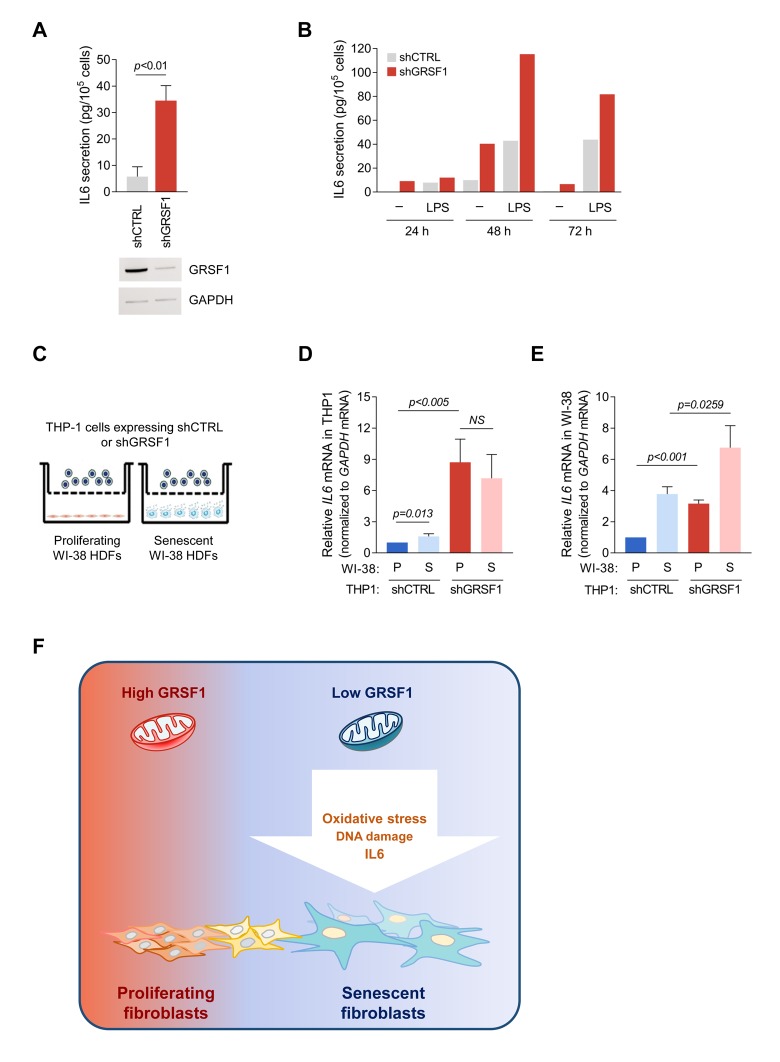
**Loss of GRSF1 increases the paracrine actions of IL6.** (**A**) THP-1 monocytes expressing lentiviral shGRSF1 or shCTRL were maintained in normal culture medium (containing 10% FBS), and the amount of IL6 secreted into the CM was measured by ELISA. (**B**) Human monocyte THP-1 cells were infected with shGRSF1 or shCTRL lentiviruses to produce different steady-state levels of GRSF1 constitutively. The levels of IL6 secretion by these cells was measured by ELISA; treatments with LPS (50 ng/mL) for 24, 48, and 72 h were included as positive controls for IL6 production. (**C**-**E**) Schematic of the co-culture setup. Proliferating (PDL20-25) or senescent (PDL55-60) WI-38 fibroblasts were plated in the lower chamber, and THP-1 cells constitutively expressing different levels of GRSF1, as explained in (**A**), were plated in the upper chamber. The chambers were separated by a selectively permeable membrane with 0.4 µm-diameter pores (**C**). Lentiviral shRNA-expressing THP-1 cells were co-cultured with either proliferating or senescent WI-38 fibroblasts for 48 h, and the levels of *IL6* mRNA in THP-1 cells (**D**) and WI-38 cells (**E**) were quantified by RT-qPCR analysis. Data in **A**,**D**,**E** represent the means ±S.D. from three independent experiments. (**F**) Model: we propose that loss of GRSF1 contributes to cellular senescence characterized by oxidative stress, DNA damage, growth suppression, and IL6 production.

Activated monocytes are the primary source of IL6 in blood, particularly during inflammation [13]. Silencing GRSF1 by stable expression of lentivirus-delivered shRNA in human THP-1 monocytes significantly elevated IL6 secretion (Fig. 5A,B). THP-1 cells treated with the IL6 inducer lipopolysaccharide (LPS) were included as a positive control (Fig. 5B). To test if IL6 produced by THP-1 cells might influence neighboring cells, we co-cultured proliferating or senescent WI-38 fibroblasts (bottom chamber) with either shGRSF1 or shCTRL THP-1 cells (top chamber) (Fig. 5C). As shown above, expression of *IL6* mRNA was significantly induced in GRSF1-depleted THP-1 cells regardless of the type of co-cultured fibroblasts (Fig. 5D). Interestingly, *IL6* mRNA levels were higher in WI-38 fibroblasts (proliferating or senescent) co-cultured with the shGRSF1 population of THP-1 cells (Fig. 5E). Collectively, these findings indicate that the increase in IL6 caused by lowering GRSF1 can promote the local production of IL6 in neighboring cells.

## DISCUSSION

Our results indicate that loss of GRSF1 triggered mitochondrial stress associated with several traits of senescence, including a DNA damage response characterized by a rise in γ-H2AX levels and 53BP1 foci, increased production and secretion of the pro-inflammatory factor IL6, and suppression of cell proliferation. Accordingly, we propose that the loss of GRSF1 observed in senescent cells contributes to a senescence-associated rise in DNA damage, oxidative stress, growth arrest, and SASP (Fig. 5F).

Mitochondrial dysfunction is one of the most widely recognized features of organismal aging and cell senescence [14], but the molecular details are poorly understood. Links between mitochondrial DNA mutations and aging are supported by several studies. Prominent examples are a knock-in mouse expressing proofreading-deficient mitochondrial DNA polymerase POLG (D257A) that exhibited premature aging [15,16] and studies showing that mitochondrial damage triggers senescence [17]. Importantly, senescent cells accumulating with age contribute to declining organ function and increased age-related disease [18]. In this regard, mouse models in which senescent cells were selectively eliminated by genetic or pharmacologic methods displayed improved age-associated conditions (e.g., cataracts, sarcopenia, osteoarthritis, atherosclerosis) [19,20]. Heterochronic parabiosis and serum transfer experiments in mice further showed that circulating factors (including SASP factors IL6, IL8, and MCP-1) may be therapeutic targets in aging. Thus, our findings that GRSF1 prevents SASP-associated IL6 production offer valuable insight into the role of mitochondria in cell senescence and organismal aging [8,21].

Initially identified as B-cell differentiation factor 1, the cytokine IL6 has multiple activities. During early phases of infectious inflammation, IL6 is produced by monocytes and macrophages following stimulation of Toll-like receptors (TLRs) by infectious or noninfectious inflammation (e.g., following traumatic injury), as well as by endothelial cells, mesenchymal cells, and fibroblasts [22]. The acute rise in IL6 expression levels stimulates various cell populations, in turn activating the host defense. Once the stress trigger is removed, the signal transduction cascade mediated by IL6 is rapidly shut off by a regulatory system that controls IL6 serum levels. However, persistent dysregulation of IL6 production (e.g., if senescent cells have accumulated in a given tissue) has been implicated in several autoimmune conditions, chronic inflammatory diseases, and cancer [23]. Given that mitochondria play a critical role in the cellular responses to tissue injury, pathogens, and inflammation, the pathologies associated with elevated IL6 may be associated with the cellular accumulation of dysfunctional mitochondria [24,25], in agreement with the aberrant IL6 production observed in THP-1 monocytes, as well as in senescent WI-38 fibroblasts expressing reduced levels of GRSF1.

In closing, given its multi-leveled impact on cell senescence, GRSF1 is emerging as an attractive target for therapeutic interventions aimed at modulating cellular senescence. At present, there are no known chemical inhibitors of GRSF1, and the molecular suppressors of GRSF1 expression, mitochondrial localization, and activity remain to be identified systematically. Likewise, virtually nothing is known about the molecular activators of GRSF1 abundance. For example, the factors that control *GRSF1* mRNA transcription, as well as the proteases that might modulate GRSF1 protein stability await further investigation. Whether these factors are responsible for reducing *GRSF1* mRNA levels (e.g., after IR) and the labile GRSF1 protein in senescent cells also remains to be investigated. A comprehensive understanding of positive and negative regulators of GRSF1 function will allow the development of GRSF1-directed tools that can improve conditions and diseases affected by senescent cells.

## MATERIALS AND METHODS

### Cell culture, establishment of senescence, transfection, treatments, and SA-β-gal activity

THP-1 cells were cultured in RPMI-1640 supplemented with 10% FBS, antibiotics, antimycotics (Invitrogen). WI-38 human diploid fibroblasts (Coriell Cell Repositories) were cultured in DMEM supplemented with 10% FBS (Gibco), antibiotics, antimycotics, and non-essential amino acids (Invitrogen). Cells were counted using a TC-20 cell counter (Bio-Rad). Proliferating WI-38 fibroblasts were used at population doubling levels (PDLs) ranging between 20-25, and senescent WI-38 fibroblasts were used after additional culture (PDL50-PDL59). WI-38 cells (PDL25) were treated with Doxorubicin (2 µg/mL) for 24 h and were harvested for analysis 7 days later. Proliferating WI-38 cells (~PDL25) were rendered senescent by exposure to 10 Gy of ionizing radiation (IR); cells were harvested 10 days later unless otherwise indicated. Cells were transfected with 50 nM of control or GRSF1-directed siRNAs (siCTRL and siGRSF1, respectively) using Lipofectamine 2000 (Thermo Fisher Scientific).

Lentiviral particles delivering shGRSF1 or shCTRL (Origene) were transduced at multiplicity of infections (MOIs) of 10-20 in the presence of 8 μg/mL of Polybrene (Santa Cruz Biotechnology). To generate THP-1 cells expressing constitutively lower levels of GRSF1, cells were transduced with lentiviral particles expressing shGRSF1 or shCTRL (Origene) one week later they were treated with puromycin (0.5 μg/mL) to select for cells stably expressing integrated vectors. Pooled GRSF1-silenced populations were used for experiments. Senescence-associated-β-galactosidase (SA-β-gal) activity was assessed using a kit (Cell Signaling).

### Reverse transcription (RT) and real-time quantitative (q)PCR analysis

Total RNA was extracted using TriPure Isolation Reagent (Roche), and cDNA was synthesized using random hexamers and reverse transcriptase (Invitrogen). Gene-specific primers were then used for qPCR amplification by employing SYBR Green master mix (Kapa Biosystems) and an Applied Biosystems 7300 instrument. The primer sequences for regular RT-qPCR analysis (each forward and reverse) were:

GCCCAGCTATGAACTCCTTCT and GAAGGCAGCAGGCAACAC for *IL6* mRNA,

GGGAAACGAAGGGGTGATG and GCGGTGCTTCTCTAAGGCTTT for *GRSF1* mRNA,

TGCACCACCAACTGCTTAGC and GGCATGGACTGTGGTCATGAG for *GAPDH* mRNA,

CATGTACGTTGCTATCCAGGC and CTCCTTAATGTCACGCACGAT for *ACTB* (β-Actin) mRNA,

CGAACGTCTGCCCTATCAACTT and ACCCGTGGTCACCATGGTA for *18S* rRNA.

### Protein preparation and Western blot analysis

Cells were lysed in modified lysis buffer (MEB) (50 mM Tris-Cl [pH 7.5], 150 mM NaCl, 1 mM MgCl_2_, and 1% NP-40) supplemented with 1 mM PMSF and a protease and phosphatase inhibitor cocktail (Thermo Fisher Scientific). Membrane proteins were isolated using the Mem-PER™ Plus Membrane Protein Extraction Kit (Thermo Scientific). To measure protein stability, WI-38 proliferating (PDL25) and senescent cells (PDL50-51) were incubated with 100 µg/mL of cycloheximide (Calbiochem) for the times indicated, then lysed for analysis. Proteins were size-fractionated by SDS-PAGE using 4-12% NuPAGE gels, transferred onto nitrocellulose membranes (iBlot Stack, Thermo Fisher Scientific). The primary antibodies used recognized were used recognizing these proteins: GRSF1 (AV40382) from Sigma; β-Actin (sc-47778), HSP90 (sc-1055), p53 (sc-126), and GAPDH (sc-32233), from Santa Cruz Biotechnology; p21 (05-345) from Millipore; TOMM20 (ab186735) from Abcam; and TOMM70 (14528-1-AP) from Proteintech. After incubation with secondary antibodies (GE Healthcare), Western blotting signals were detected by enhanced chemiluminescence (Millipore) using a KwikQuant Imager (Kindle Biosciences, LLC).

### Immunofluorescence analysis

Fixed or live cells were imaged using a confocal microscope LSM710 (Carl Zeiss, Inc.) or KEYENCE fluorescence microscope (BZ-X700). Immunofluorescence was performed as previously described [9]. Briefly, WI-38 fibroblasts were fixed with 100% methanol (Fisher Scientific) or 3.7% formaldehyde (methanol-free, Polysciences) for 10 min at -20 °C or RT, respectively, and permeabilized in 0.2% Triton X-100 for 5 min. After washes with PBS and incubation with 10% normal goat serum (Thermo Fisher Scientific) for 16 h at 4 °C, anti-GRSF1 (Sigma) or anti-53BP1 (BD Biosciences) antibodies were added to the cells and incubated at 37°C for 1 h. Washed cells were then incubated at 37 °C for an additional 30 min with Alexa Fluor 568-conjugated secondary antibodies (Thermo Fisher Scientific). Nuclei were stained in ProLong® Gold Antifade reagent with DAPI (for fixed cells, Thermo Fisher Scientific) or Hoechst 33342 dye (for live cells, Invitrogen). To measure the formation of mitochondrial superoxide, WI-38 fibroblasts were stained with a fluoroprobe (MitoSOX Red, Thermo Scientific) and assessed by the Keyence Fluorescence Microscope (Keyence).

### Flow cytometry

To monitor DNA double-strand breaks, fluorescently-labeled γH2AX was detected by flow cytometry. Briefly, cells were fixed for 1 h at 25 °C, washed, and permeabilized (00-8333-56, eBioscience). After one wash with FACS buffer (0.5% BSA in PBS), cells were incubated with mouse control IgG-PE (BioLegend) or anti-H2AX(pS139)-PE (BD Biosciences) in the dark for 1 h at 25 °C. Washed cells were transferred to FACS tubes for analysis by flow cytometry (FACSCanto II, BD Biosciences).

### [^3^H]-Thymidine incorporation assay

WI-38 fibroblasts expressing lentiviral shRNAs (shGRSF1 or shCTRL) were cultured in 6-well plates and incubated with 1 µCi/mL of [^3^H]-thymidine (Perkin Elmer) for 16 h at 37°C, whereupon cells were washed twice with ice-cold PBS, washed twice with 5% TCA, and solubilized in 0.8 mL lysis buffer (0.5 M NaOH, 2% SDS). The solubilized cell suspension (0.4 mL) was collected into vials containing 5 mL of scintillation cocktail and the radioactivity was measured using LS6500 scintillation counter (Beckman). Data were normalized to total protein amounts used for each sample.

### Measurement of secreted IL6

The AlphaLISA® Human Interleukin-6 (IL6) Detection Kit (Perkin Elmer Inc.) was used to measure IL6 in CM following the manufacturer's instructions. To prepare conditioned media (CM), cells were cultured in serum-depleted media overnight, unless indicated. IL6 levels were normalized the number of cells in the plate. For ELISA, CM was prepared as follows: cells were washed with 1× PBS twice, then incubated with fresh DMEM with 10% FBS. After incubation for an additional 24-48 h, the CM was collected for further analysis. IL6 secreted into the culture medium were measured using a Human IL6 Quantikine ELISA Kit (R&D systems).

### Polysome analysis

Polysome analysis was performed as previously described [26]. Briefly, cells were treated with cycloheximide (Calbiochem; 100 µg/mL) for 10 min and lysed in PEB (polysome extraction buffer; 20 mM Tris-HCl [pH 7.5], 100 mM NaCl, 5 mM MgCl_2_ and 0.3% NP-40). After the lysate was separated through 10% to 50% sucrose gradients, 12 fractions were collected for further analysis. The distribution of mRNAs was quantified by RT-qPCR analysis and plotted as a percentage of the specific mRNA in each fraction relative to the total amount of that mRNA in the gradient.

### Cell synchronization by double thymidine block and FACS analysis

To block cell cycle progression, GRSF1-silenced (shGRSF1) and control (shCTRL) WI-38 fibroblasts were incubated with 2 mM thymidine (Sigma Aldrich) for 18 h at 37 °C and then washed with PBS twice and incubated in standard culture media for an additional 9 h; cells were then cultured in thymidine-containing media for an additional 15 h to achieve synchronization. Synchronized cells were washed twice with PBS, and after adding fresh media to release cells into the division cycle, cells were collected after varying time periods. For collection, cells were trypsinized, resuspended and fixed in 70% ethanol, and kept at -20°C for 16 h. One day later, cells were pelleted and resuspended in PBS, propidium iodide (PI) was added to stain the DNA, and cell cycle analysis was performed using a Canto II flow cytometer (BD Biosciences) and FlowJo Software (FlowJo v10.2).
